# Significantly delayed polyglactin 910 suture-related pseudoinfection in a Yucatan pig

**DOI:** 10.1186/s12917-020-02662-3

**Published:** 2020-11-25

**Authors:** Dalis Collins, Brian Simons

**Affiliations:** Center of Comparative Medicine (DC) and Diagnostic Pathology Laboratory (BS), Baylor College of Medicine, Houston, TX 77030 USA

## Abstract

**Background:**

Polyglactin 910 is a synthetic braided, absorbable suture commonly used in surgery. Though polyglactin 910 suture-related pseudoinfection is well documented in the human literature, it has not been previously reported in the veterinary literature.

**Case description:**

A 3-year-old female, ovariectomized but otherwise experimentally naïve Yucatan pig was evaluated for a several week history of bilateral multifocal abscesses in the area of the paralumbar fossa, which continued to worsen despite oral antibiotics and non-steroidal anti-inflammatory medications. The multi-focal abscesses continued to worsen and additional diagnostics were pursued including cytology, culture (aerobic, anaerobic and fungal), and bloodwork. All supported a non-infectious etiology. Biopsy results indicated a suture-related pseudoinfection. Despite treatment including parenteral antibiotics, pain medications and superficial surgical debridement, the dermatologic lesions worsened. Euthanasia was elected. Post-mortem necropsy demonstrated a suture-related pseudoinfection with extrusion of suture material from the ovarian pedicle ligatures through the body wall and skin leading to numerous sterile abscesses in the bilateral paralumbar fossa.

**Conclusions:**

This is the first published report of a significantly delayed polyglactin 910 suture-related pseudoinfection in a Yucatan pig. While likely an isolated incident, it supports further research into this area. Additionally for critical research studies using Yucatan pigs, pre-surgical assessment with hypersensitivity patch testing may be appropriate.

## Background

Even with the advent of new biomaterials and the switch to synthetic suture for many applications, acute post-operative suture reaction still occurs. However, suture-related pseudoinfection (SRPI) is a less common condition and may be misidentified due to delay of onset. SRPI is characterized by local or systemic signs of inflammation occurring approximately 6–9 weeks after suture placement.

There are few case reports in the veterinary literature of a foreign body reactions to polyglactin 910. One study compared polyglactin 910 to two other suture types in the immediate post-surgery period (10–14 days) when used in the linea alba closure of cats for ovariohysterectomy [1]. The least reaction or inflammation in this study occurred with a polyglactin 910 closure without additional closure of the subcutaneous tissues. Another study comparing inflammation secondary to suture incisional closure in rats found that Maxon® (polyglyconate) and PDS® (polydioxanone) elicited a lower degree of chronic inflammation compared with Vicryl® (polyglactin 910) and chronic gut. Overall healing times were comparable [2]. These reports therefore support polyglactin 910 as a safe and appropriate suture choice in many situations based on clinical signs [1] and histology [1, 2]. However, there has been little published on polyglactin 910 suture with regards to safety and immunogenicity in the veterinary literature since the 1980 s.

## Case presentation

A 3 year old adult female Yucatan pig acquired from an approved research vendor^1^ arrived healthy to the research facility. The pig was never enrolled into the study for which it was ordered, and at the time, there were no concurrent studies to allow transfer to another experimental protocol. Therefore, the experimentally naive animal was then transferred to the institutional holding protocol. For the purposes of interim group housing and future re-homing or adoption, a bilateral ovariectomy was performed via a midline laparotomy (~ 20 cm incision length). The animal was anesthetized with Telazol^2^ (4 mg/kg IM) and Xylazine^3^ (2 mg/kg IM) and maintained intubated with isoflurane anesthesia^4^ (2–3%). The surgical site was prepared with removal of all hair and a triple surgical scrub using chlorohexidine and alcohol. Peri-operative cefazolin^5^ (20 mg/kg, IV, once) was administered, and aseptic surgical technique was maintained throughout the procedure. 0-Vicryl® (polyglactin 910)^6^ miller knots were used for ligation of each ovarian pedicle. For added security, each pedicle was double ligated in this manner. Three-layer closure of the incision was accomplished using PDS II® (polydioxanone)^6^ in a simple continuous pattern (size 2 for linea alba, size 0 for subcutaneous tissue, and size 2 − 0 for intradermal). Pre-operative analgesia was provided with Buprenorphine SR^7^ (0.2 mg/kg SQ once), meloxicam^8^ (0.4 mg/kg, SQ, once), and a local incisional block (5 mL 2% Lidocaine^9^). Post-operatively carprofen^10^ (4 mg/kg, PO, q24) was provided for 3 days. The surgery and post-operative period were uneventful.

Approximately 4 months later (122 days post-surgery), a small nodule (~ 1 cm in diameter) developed on the left paralumbar fossa with the surrounding 3–4 cm of skin warm and mildly hyperemic. Fine need aspirate of the nodule revealed clear serous fluid. Cytology did not reveal any cellular infiltrates and protein assessment was not performed at that time. All other physical exam findings were unremarkable. A presumptive diagnosis of a cyst or seroma was made based on these findings.

**Fig. 1:**
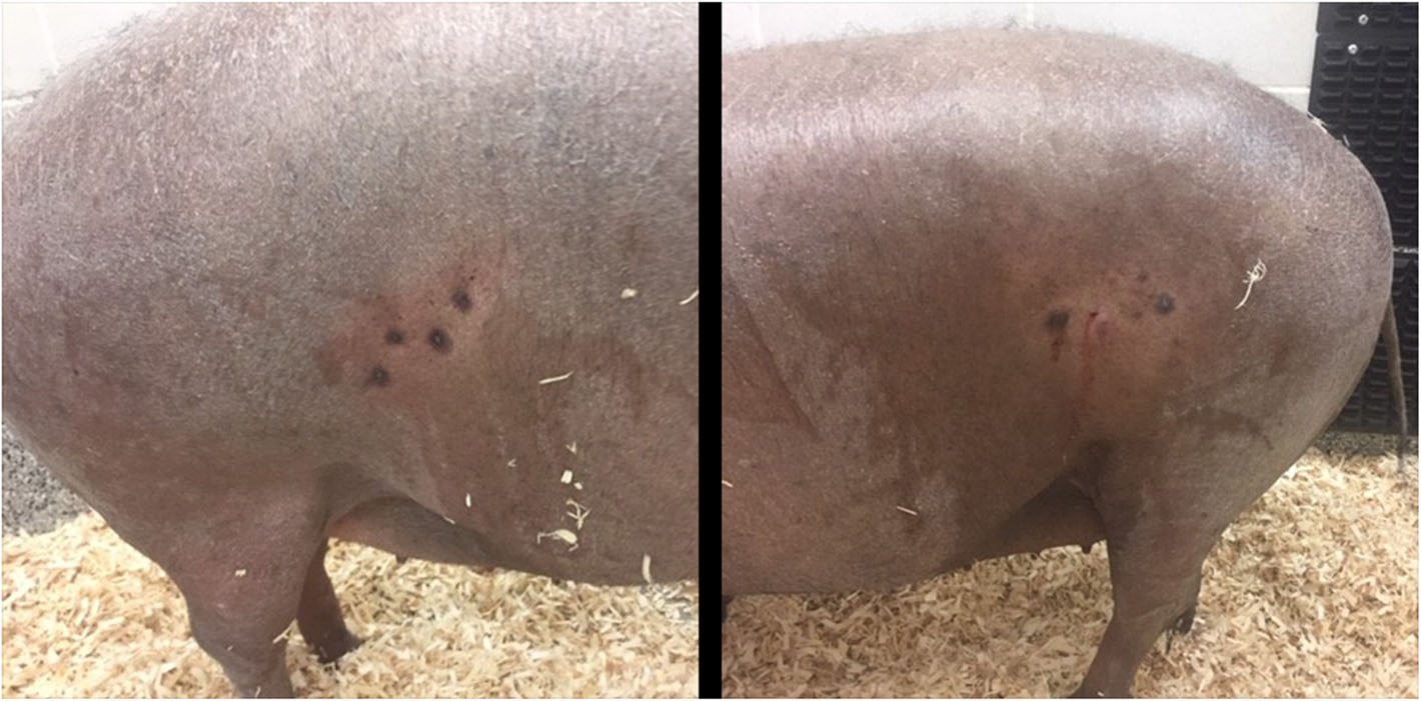
Gross presentation of initial clinical signs present on left and right paralumbar fossa. Numerous multifocal nodules and draining wounds present measuring x < 2.5 cm in diameter

By one month after this (146 days post-surgery), both paralumbar fossa had developed 2–5 nodules present on each side. Fine need aspirate yielded caseous material indicating abscessation. In-house cytology demonstrated numerous degenerate neutrophils admixed with cellular debris. No bacteria, fungi, or parasites were identified on cytology. Due to worsening presentation, treatment including amoxicillin^11^ (15 mg/kg, PO, q12) and carprofen^10^ (4 mg/kg, PO, q24) were instituted. Despite two weeks of treatment, there was no improvement and the affected area on each side measured 8 cm x 8 cm with multifocal abscesses present. The surrounding area was still warm and mildly hyperemic, and the immediate area was becoming hyperpigmented (Fig. 1).

Due to this progression, anesthetized debridement, bloodwork, culture, and cytology were elected (166 days post-surgery). The animal was anesthetized as previously described. An intravenous catheter was placed in the right auricular vein and cefazolin^5^ (20 m/kg, IV, q12) and enrofloxacin^12^ (5 mg/kg, IV, q24) started. These antibiotics were continued for four days after debridement. Oral carprofen therapy was continued throughout this period. All abscesses were sterilely lanced and flushed with dilute betadine and sterile saline and allowed to heal open. Aerobic, anaerobic, and fungal cultures were submitted to an external laboratory^9^, but all yielded no growth. Cytology taken at time of debridement was unchanged and characterized by neutrophilic inflammation. A complete blood count and serum chemistry was performed on in-house analyzers.^10^ Complete blood count results indicated a chronic inflammatory process with a mild non-regenerative anemia and a shift in the leukocyte percentages with neutrophils comprising 73% of the total white blood cells and lymphocytes only 22% (Table 1). The elevated creatinine kinase is attributable to a traumatic blood collection. The low sodium was determined to be spurious secondary to a needed machine maintenance for that particular analyte (Table 2). All other complete blood count total numbers and serum chemistry values were within normal ranges.

**Table 1:**
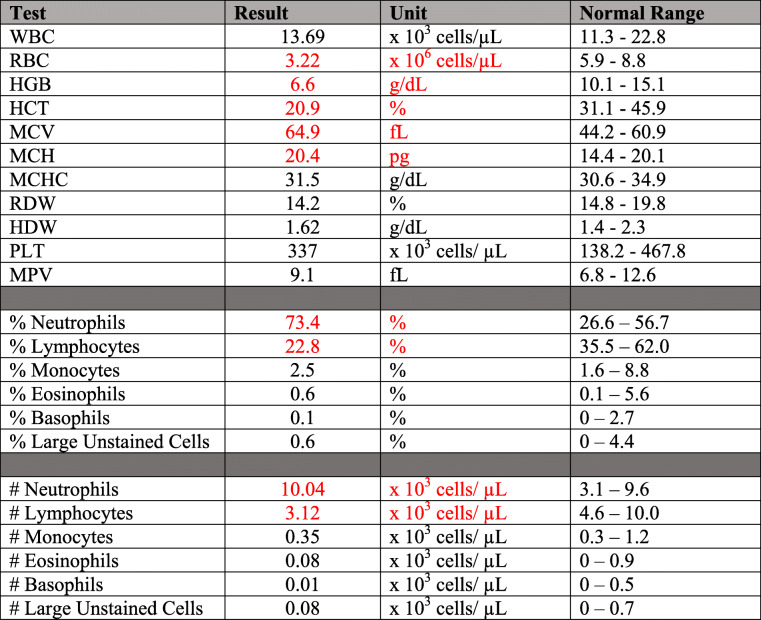
Complete blood count. Results outside normal ranges as provided by the vendor for age and sex matched control animals are highlighted in red

**Table 2:**
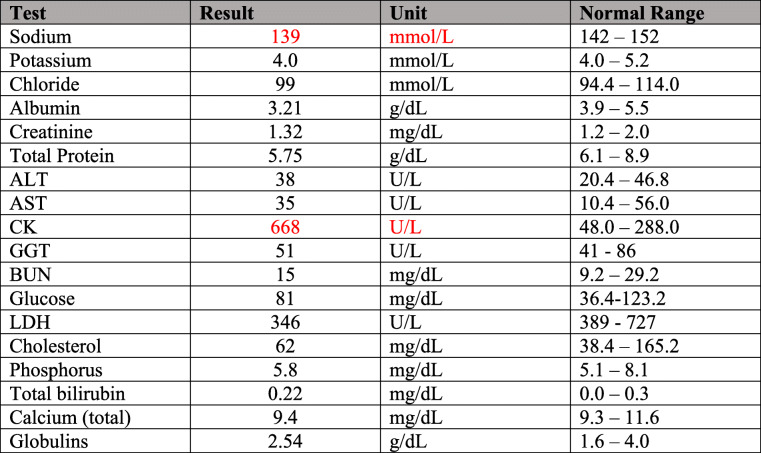
Serum chemistry values. Results outside normal ranges as provided by the vendor for age and sex matched control animals highlighted in red

**Fig. 2:**
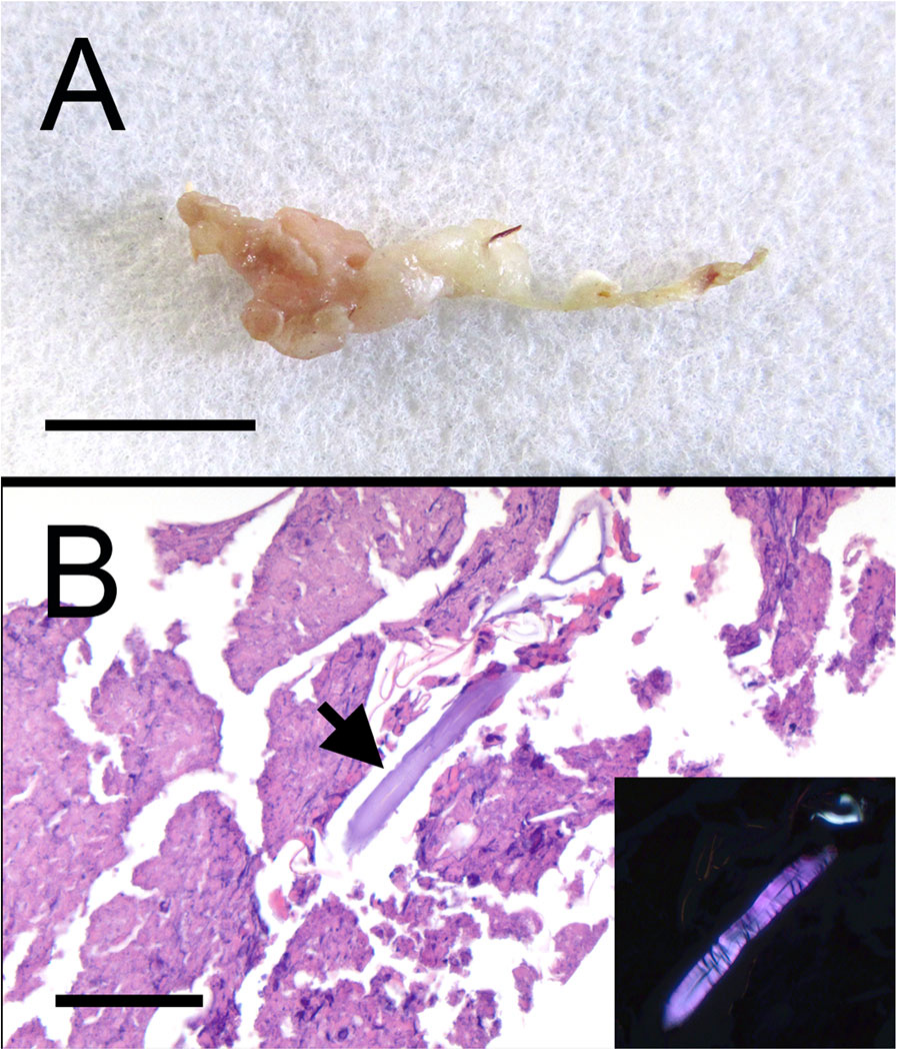
**a** Gross presentation of material removed from one of the draining tracts in the paralumbar fossa. Scale bar, 1 cm. **b** Low magnification view of the foreign material surrounded by subcutaneous tissue. (H&E, bar = 50 µm) Inset of the same image under polarized light highlights the refractile nature of the foreign material

Approximately 2 weeks after the surgical debridement during cleaning of the area (177 days post-surgery), a small biopsy was collected from one of the draining tracts which included fibrous material (Fig. 2a). Histologically, foreign material was identified in the core of the tissue (Fig. 2b). Reviewing the animal’s record, it was concluded at the observed lesions were likely a result of a reaction to the pedicle ligatures made with 0-Vicryl® during the ovariectomy. No other foreign materials had been introduced to the animal during its ovariectomy or any time thereafter.

**Fig. 3:**
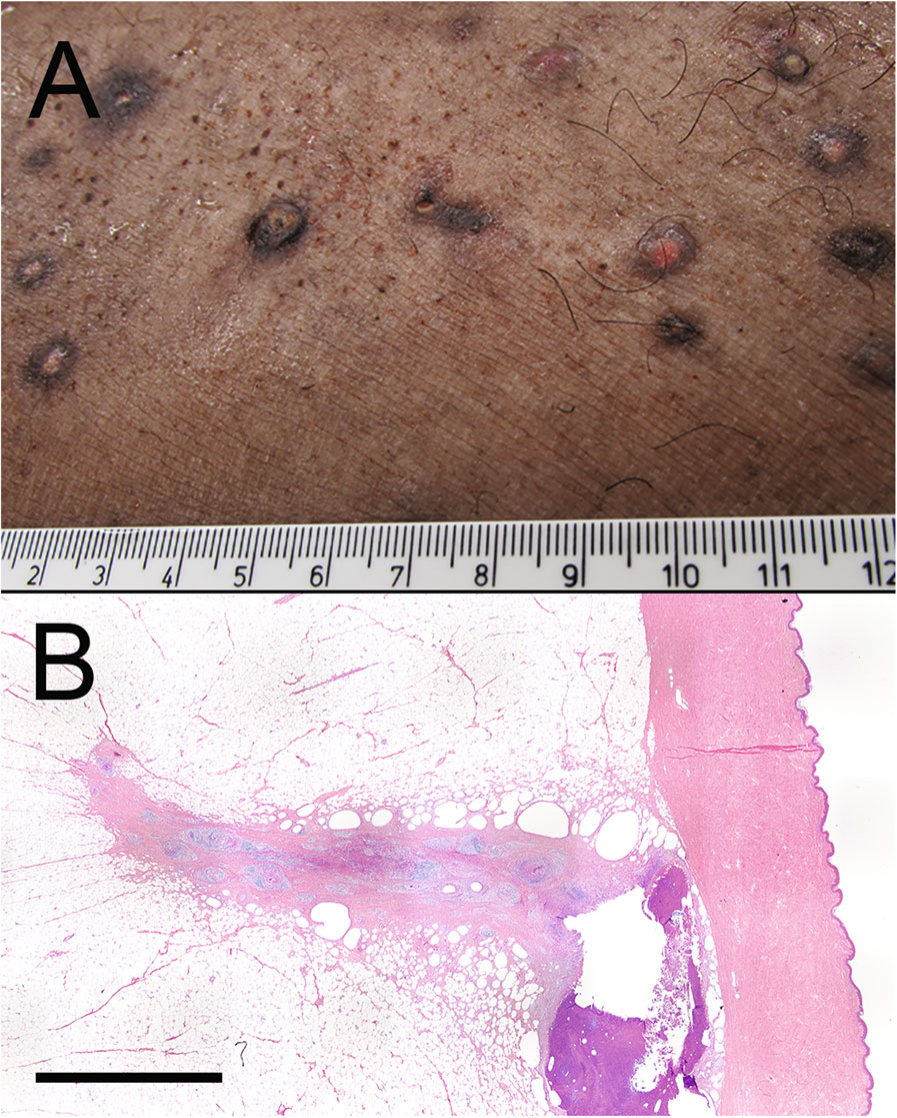
Images and histology taken from post-mortem necropsy. **a** Gross pathology present from a piece of tissue from the right paralumbar fossa. **b** Low magnification view of histology demonstrating presence of draining tracts in the subcutaneous tissue. (H&E, bar = 5 mm)

As a suture-related pseudoinfection causing a sterile inflammatory process, all antibiotics were discontinued, and the animal was maintained on intermittent carprofen^10^ (4 mg/kg, PO, PRN) and buprenorphine HCl^13^ (0.03 mg/kg, SQ, PRN) to control pain and discomfort. Further, more invasive surgical debridement was not elected. Despite supportive care, the animal developed intermittent mild hindlimb lameness which alternated limbs depending on which paralumbar fossa was more severely affected with abscesses and draining tracts at the time. Eventually, the animal became generally more agitated and aggressive to care takers. Because of this change in behavior and lack of response to treatment, euthanasia was elected approximately 5 months after initial presentation and 9 months after ovariectomy. For euthanasia, similar sedation was used as for debridement, and the animal euthanized (Beuthanasia-D®, 4.5 mg/kg, IV). At necropsy (241 days post-surgery), numerous abscesses ranging from 3 to 7 mm diameter were present bilaterally in the skin and subcutaneous fat (Fig. 3a). These lesions occasionally extended in a linear fashion deep into the subcutis and through the body wall (i.e. draining sinus) (Fig. 3b). Ovarian pedicles were bilaterally fibrotic and mineralized with linear tracks of fibrotic tissue connecting the ovariectomy site with skin lesions. Minor abdominal adhesions were multifocally present (i.e. post-surgical adhesions). While scarring was present from the linea alba closure, there was no indication grossly of a similar foreign body reaction or remaining suture from the surgery in that area. Tissue sections were fixed with 10% formalin, paraffin embedded, and sectioned for hematoxylin and eosin staining.

**Fig. 4:**
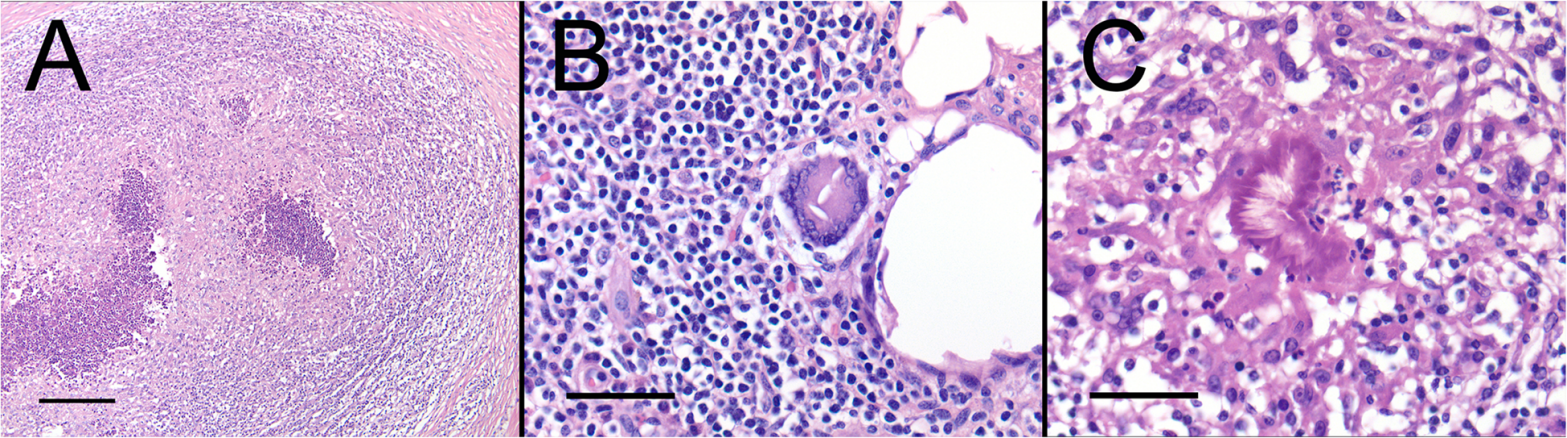
Characteristic histopathology of a suture-related pseudoinfection present in this animal. **a** Low magnification view showing extensive areas of pyogranulomas. (H&E, bar = 200 µm) **b** Higher magnification view of an area of pyogranuloma highlighting distinct multi-nucleated giant cells surrounding foreign material. (H&E, bar = 50 µm) **c** Higher magnification view of an area of inflammation which contains an aggregate of antigen-antibody complexes (Splendore-Hoeppli bodies). (H&E, bar = 50 µm)

Histologically, there were typical signs associated with a foreign body reaction including multifocal small pyogranulomas (Fig. 4a). These were observed throughout the skin which consisted of a necrotic core containing degenerate neutrophils, epithelioid macrophages and numerous foreign body type giant cells surrounded by fibroplasia and chronic inflammatory cells. In a few sections, distinct multi-nucleated giant cells surrounded a refractile material (Fig. 4b). In some of these pyogranulomas, the center contained Splendore-Hoeppli bodies composed of antigen antibody complexes (Fig. 4c).

## Discussion and conclusions

Conversely, in the human medical literature, hypersensitivities and pseudoinfections to polyglactin 910 and its derivatives are well reported for a variety of procedures. Several authors of case reports or studies state subjectively that reactions to this type of suture may be on the rise [3, 4]. In review of one doctor’s surgical records from 1999 until the early in 2005, Vicryl® was widely used for a variety of ambulatory procedures with no documented complications. However, in 2005, 11 cases of unexpected tissue reactions to Vicryl® or Vicryl-Plus® were documented [4]. Other case reports of polyglactin 910 suture extrusion include surgical manipulations such subcuticular closures after orthopedic procedures [5], hip arthroscopies [6, 7], and facial plastic surgery [5]. In a retrospective medical study evaluating sclerotomy closure using a variety of suture types, post-operative adverse suture reaction rates were 2% for plain gut, 30% for polyglycolic acid, and 12% for polyglactin 910 [8].

In some case reports, there is speculation on if there is a requirement for an interaction with another substance such as Dermabond® [4] or tuberculin [5]. These case reports may indicate that for some patients the use of Vicryl® alone may not sufficient to induce a reaction, and other factors are needed. For the case report described here, besides an adhesive bandage^14^ applied to cover the incision site in the immediate post-operative period, the animal was not exposed to any other foreign substances (vaccines, dermal adhesive, etc.) the 4 months prior or after the ovariectomy. In anticipation of a potential complication, some medical doctors now use patch testing based using a published visual [3] or histologic [9] grading system to evaluate sensitivity prior to use of Vicryl® in their patients prior to elective cosmetic procedures. However, the feasibility of this in a veterinary or laboratory animal setting is questionable.

Significant delays, up to years, after initial surgery have been documented for suture-related pseudoinfections or other reactions for silk [10–12]. As a braided non-absorbable suture made of natural materials, this is not surprising. However, polyglactin 910 is a braided synthetic absorbable suture. While suture reactions often become apparent in the immediate weeks after surgery, there was a 4–5 month delay from time of surgery until the initial presentation. Drake et al. studied subcutaneous suture reaction in Gottingen mini-pigs comparing extrusion rates over a 5 week period from the lateral aspect of the hindlimb and abdomen. Coated Vicryl® had a significantly higher extrusion rate than that of Polysorb® (31% vs. 19%) in this study [13]. Both this case study and the aforementioned research study appear to contradict the package insert which states that Vicryl® absorption is typically complete in 56–70 days [14].

The package insert also warns of suture extrusion and delayed absorption in areas of poor vascular supply [14]. Fat has an extremely limited vascular supply compared to other tissues. In this animals, there was extensive intra-abdominal fat in the peri-ovarian area, some of which was likely enclosed in the pedicle tie or in close approximation to the suture ends. Additionally, pigs are notable animal models for obesity and fat deposition. Therefore, pigs may be at a higher risk of suture extrusion due to more extensive fat deposits. Reabsorption rates and likelihood of reaction are also directly correlated to the amount and size of suture used. Drake et al. also concluded secure knot formation using the least amount of material should be a priority to reduce the amount of suture left in the body with an significantly higher extrusion rates for 5-throw surgeon’s knots versus 3-throw or 4-throw [13]. Unfortunately, electrocautery was not available for use during this animal’s ovariectomy, and the amount of suture used to perform the double ligation miller pedicle ties in addition to the large suture size may have predisposed this animal.

It is important to note that polyglactin 910 suture-related pseudoinfection is not at this time considered a significant post-operative complication for pigs. This is evidenced by the numerous pig surgeries performed every year at research and clinical institutes using this suture type in addition to recent publications outlining surgical complications in pet pigs of which this was not a major finding [15, 16]. More research is needed to determine the contribution of individual hypersensitivity in the presented cases. While an exact cause cannot be determined for the suture-related pseudoinfection presented here, there are several important clinical points that this case highlights. Suture-related pseudoinfection should be considered as a differential for any animal that develops multifocal abscesses overlying a prior surgical area if no infectious agent can be identified. Additionally, as part of a full diagnostic work up, early biopsy in non-healing dermal lesions is highly recommended and may reveal an underlying etiology much earlier than in the presented case.

## Data Availability

All data generated or analyzed during this study are included in this published article.

## Abbreviations

PDS: Polydioxanone
SRPI: Suture-related pseudoinfection

## Footnotes

1 Sinclair Research Inc., Auxvasse, MO

2 Zoetis, Parsippany-Troy Hills, NJ

3 AnaSed, Akorn Pharmaceuticals, Lake Forest, IL

4 Isothesia, Henry Schein, Melville, NY

5 Hikma Pharmaceutical, London, United Kingdom

6 Ethicon Inc., Somerville, NJ

7 Zoopharm, Laramie, WY

8 Noorbrook Laboratories Ltd, Newry, Northern Ireland

9 Henry Schein, Melville, NY

10 Putney Inc, Portland, MO

11 Hospira, Lake Forest, IL

12 Baytril, Bayer, Leverkusen, Germany

13 Buprenex, Reckitt Benckiser, Hull, England

14 Siemens Advia 120, Munich, Germany and Beckman Coulter AU480, Pasadena, CA
